# CCL17‐CCR4 axis contributes to the onset of vitiligo in mice

**DOI:** 10.1002/iid3.423

**Published:** 2021-06-02

**Authors:** He Li, Congpin Wang, Xiaoqing Li, Yinghui Kong, Weiguo Sun

**Affiliations:** ^1^ Department of Dermatology The Affiliated Huaian No. 1 People's Hospital of Nanjing Medical University Huai'an China; ^2^ Department of Pharmacy, Eye Ear Nose & Throat Hospital Fudan University Shanghai China

**Keywords:** CCL17, CCR4, CD8^+^ T cells, neutralizing antibody, vitiligo

## Abstract

**Background:**

Destruction of melanocytes mediated by autoimmunity is currently believed as the main cause of vitiligo. This article aims to identify the role of CC chemokine ligand 17 (CCL17)–CC chemokine receptor 4 (CCR4) axis in vitiligo and provide new possibilities for the clinical treatment of vitiligo.

**Methods:**

A total of 30 patients with vitiligo from Affiliated Huaian No. 1 People's Hospital of Nanjing Medical University were recruited based on the inclusion and exclusion criteria. Trephine was used to obtain skin samples from the lesion area and its surrounding normal areas, and the expression levels of CCL17, CCR4, Tbx21, Eomes, and Blimp1 were determined by quantitative reverse transcription polymerase chain reaction. Vitiligo mouse model was established by adoptively transferring CFP‐PMEL CD8+ T cells into sublethally irradiated Krt14‐Kitl* mice. Recipient mice received intraperitoneal injection of 1 × 10^6^ plaque‐forming units of rVV‐hPMEL on the same day of transfer. The degree of depigmentation was scored blindly by one observer 5 weeks after vitiligo induction. CFP‐PMEL CD8+ T cells migration to skin, draining lymph nodes, spleen, and blood were detected by flow cytometry. CCR4 blockade was performed by intraperitoneal injection of neutralizing antibody.

**Results:**

The expression levels of CCL17, CCR4, Tbx21, Eomes, and Blimp1 in skin lesions were significantly increased compared with that in surrounding normal areas. CCL17^−/−^ and CCR4^−/−^ mice exhibited significantly lower disease scores than WT mice. The CFP‐PMEL CD8+ T cells accumulation was significantly decreased in the skin of CCL17^−/−^ and CCR4^−/−^ mice, but was not changed in draining lymph nodes, spleen, and blood. Administration of CCR4 neutralizing antibody decreased the degree of depigmentation and the recruitment of CFP‐PMEL CD8+ T cells to the skin, while keeping the number of T cells in draining lymph nodes unchanged.

**Conclusion:**

Targeting CCL17‐CCR4 axis might inhibit T cell migrating to skin and alleviate vitiligo progression.

## Graphical abstract

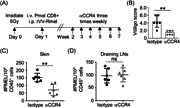

## 1 INTRODUCTION

Vitiligo is clinically depigmented skin disease which incidence rate is between 0.1% and 2%.^1^ The skin lesions appear as milky white patches or gray hairs with clear boundaries and appear on the skin, mucous membranes, or hair.^2^ Vitiligo seriously affects the mental health and life quality of patients, and treatment is relatively difficult.^3^ According to the clinical manifestations of skin lesions, vitiligo could be divided into segmental, nonsegmental, mixed, and undefined types.^4^ The etiology of vitiligo is complicated. Hypotheses about its pathogenesis include autoimmune theory, genetic theory, neurology, oxidative stress theory, and melanocyte self‐destruction theory.^5^

Effector T cells includes regulatory T (Treg) cells, CD4^+^ T helper (Th) cells, and CD8^+^ cytotoxic T cells.^6^ Recently, CD8^+^ T cell function in vitiligo pathogenesis has become a research hotspot.^7^ Some scholars have demonstrated that CD8^+^ T cells infiltration around the skin lesions has increased significantly in patients with vitiligo, which is related to the destruction of melanocytes.^8^, ^9^ Therefore, preventing CD8^+^ T cell infiltration in skin lesion area may be an important strategy for the treatment of vitiligo.

CCR4 is a CCR family member which belongs to G protein‐coupled receptors.^10^ It has 7 transmembrane fragments and could bind to two chemokines CC chemokine ligand 17 (CCL17) and CCL22 with high affinity.^11^ Studies have detected CCR4 expression in kinds of T cell subgroups, such as Treg cells and type 2 Th cells.^12^ CCR4 participates in T cell homing.^13^ Zhang et al. observed enhanced CCR4 expression in CD8^+^ T cells of patients with active vitiligo.^14^ In addition, CCL22 inhibits depigmentation and promotes Treg cell migration in vitiligo.^15^ Animal experiments on vitiligo mice have shown that upregulating the mouse chemokine CCL22 to activate the mouse Treg cells could increase the abundance of Tregs in the skin and improve the skin lesions of vitiligo.^15^, ^16^ However, no studies have reported the function of another CCR4 ligand CCL17 in regulating CD8^+^ T cells homing to skin in vitiligo. This article aims to identify the relationship between CCL17‐CCR4 axis and vitiligo and provide new possibilities for the clinical treatment of vitiligo.

## 2 METHODS

### 2.1 Patients and samples

This study was approved by the ethics commitment of the Affiliated Huaian No. 1 People's Hospital of Nanjing Medical University. All selected patients signed an informed consent form.

Inclusion criteria: 14–45 years old; the clinical diagnosis meets the classification criteria of the pigment disease group of the Dermatology and Venereology Professional Committee of the Chinese Integrative Medicine Society (annual version); existing leukoplakia enlargement or new rash within 1 year; none severe systemic diseases such as high blood pressure and heart disease; untreated patients or have not received immunosuppressive immunomodulatory system therapy at least 4 weeks before collecting materials and have not received topical drug treatment within 2 weeks; and volunteer to participate.

Exclusion criteria: female patients during pregnancy or lactation; patients with scar constitution; those who are allergic to anesthetics; those who are psychologically and physiologically unsuitable for the process of obtaining materials.

All tissue specimens are trephine, close to the edge of the leukoplakia, and cut the tissue at the edge of the leukoplakia.

### 2.2 Mice

KRT14‐Kitl*4XTG2Bjl (Krt14‐Kitl*) mice (009687), PMEL TCR mice (B6.Cg‐Thy1a/Cy Tg(TcraTcrb)8Rest/J, 005023), CCR4^−/−^ mice (B6;129P‐Ccr4tm1Pwr/J, 004101) and CAG‐ECFP mice (B6.129(ICR)‐Tg(CAG‐ECFP)CK6Nagy/J, 004218) were obtained from The Jackson Laboratory. CCL17^−/−^ mice were purchased from the Shanghai Model Organisms (NM‐KO‐190010). To generate CFP‐PMEL TCR transgenic mice, CAG‐ECFP mice were crossed with PMEL TCR transgenic mice. To generate CCR4^−/−^ CFP‐PMEL TCR transgenic mice, CCR4^−/−^ mice were crossed with CFP‐PMEL TCR mice. CCL17^−/−^ mice were crossed with Krt14‐Kitl* to generate CCL17 deficient recipients. Heterozygous Krt14‐Kitl* were applied in all experiments. Mice are kept in specific pathogen‐free facility at constant temperature and humidity, under 12h‐12h light and dark cycle. Animal studies were approved by the ethics commitment of the Affiliated Huaian No. 1 People's Hospital of Nanjing Medical University.

### 2.3 Vitiligo induction and CCR4 neutralization

Vitiligo was induced through adoptive transfer of PMEL CD8^+^ T cells. PMEL CD8^+^ T cells were isolated from the spleens of PMEL TCR transgenic mice by FACS. Purified CD8^+^ T cells (1 × 10^6^) were injected intravenously into sub‐lethally irradiated Krt14‐ Kitl* hosts. Recipient mice received intraperitoneal injection of 1 × 10^6^ plaque‐forming units of rVV‐hPMEL on the same day of transfer. To measure the migration efficiency of CCR4^−/−^ T cells to the skin within the same receipt mouse, equal numbers (0.5 × 10^6^) of GFP^+^ WT or CCR4^−/−^ T cells were injected intravenously.

CCR4 blockade was performed by intraperitoneal injection of 100 μg of neutralizing antibodies three times weekly for the duration of the observation period. Vitiligo score was objectively quantified by an observer blind to the experimental groups, using a point scale based on the extent of depigmentation at four easily visible locations, including the ears, nose, rear footpads, and tails as described previously. Each location was examined, and the extent of depigmentation was estimated as a percentage of the anatomic site; both left and right ears and left and right rear footpads were estimated together and therefore evaluated as single sites. Points were awarded as follows: no evidence of depigmentation (0%) received a score of 0, >0%–10% = 1 point, >10%–25% = 2 points, >25%–75% = 3 points, >75% to <100% = 4 points, and 100% = 5 points. The “vitiligo score” was the sum of the scores at all four sites, with a maximum score of 20 points.

### 2.4 RNA extraction and qRT‐PCR analysis

RNeasy Mini Kit (Qiagen, 74104) was applied to extract total RNA, which was transcribed to cDNA by FastKing RT Kit (With gDNase) (KR116; TIANGEN). QRT‐PCR analysis was performed by FastKing One Step RT‐qPCR Kit (SYBR Green) purchased from (FP313; TIANGEN). The primers used are shown as follows.

CCL17‐F: 5′‐CGAGAGTGCTGCCTGGATTACT‐3′

CCL17‐R: 5′‐GGTCTGCACAGATGAGCTTGCC‐3′;

CCR4‐F: 5′‐GGACTAGGTCTGTGCAAGATCG‐3′

CCR4‐R: 5′‐TGCCTTCAAGGAGAATACCGCG‐3′;

Tbx21‐F: 5′‐CCACCTGTTGTGGTCCAAGTTC‐3′

Tbx21‐R: 5′‐CCACAAACATCCTGTAATGGCTTG‐3′;

Eomes‐F: 5′‐CCACTGGATGAGGCAGGAGATT‐3′

Eomes‐R: 5′‐GTCCTCTGTCACTTCCACGATG‐3′; and

Blimp1‐F: 5′‐AAGACGTTCGGTCAGCTCTCCA‐3′

Blimp1‐R: 5′‐CTGGCACTCATGTGGCTTCTCT‐3′.

### 2.5 Flow cytometry

Mouse tissues were harvested and processed as previously described.^17^ Briefly, tail skin and draining lymph nodes were harvested at the indicated times. Lymph nodes were disrupted, and tail skin was incubated with Dispase II (5 U/ml; Roche) for 1 h at 37°C. Epidermis was removed and mechanically dissociated using 100‐μm filters. Dermis was incubated with collagenase IV (1 mg/ml) and deoxyribonuclease I (2 mg/ml; Sigma‐Aldrich) for 1 h at 37°C before mechanical dissociation. Samples were filtered before staining and analysis, and UltraComp eBeads (eBioscience) were used for compensation controls. The mouse spleen was aseptically separated, placed in a plate containing an appropriate amount of sterile PBS, and the spleen was cut into pieces with ophthalmological scissors, crushed and filtered with a 100 μm sieve, to make a single cell suspension. Centrifuge at 1000 r/min for 5 min at room temperature, discard the supernatant and add three times the cell volume of red blood cell lysate, mix and lyse for 30 s, then centrifuge and wash with PBS three times, and resuspend in RPMI‐1640 complete medium.

Flow cytometry of different tissue cell suspensions was performed by FACSAria (BD Bioscience). Antibodies used in these experiments were shown here: PB‐conjugated anti‐CD4, APC‐CY7‐conjugated anti‐CD8, APC‐conjugated anti‐CD11b, PerCP5.5‐conjugated anti‐CD45, and PE‐conjugated anti‐CD45. All the antibodies were purchased from eBioscience.

### 2.6 Statistical analysis

GraphPad Prism (version 6.01) was used for statistical analysis and data were shown as mean ± SEM. One‐way analysis of variance analysis was used to examine statistical analysis. Statistically significant was accepted as *p* < .05.

## 3 RESULTS

### 3.1 Key chemokine pathways in vitiligo

**Figure 1:**
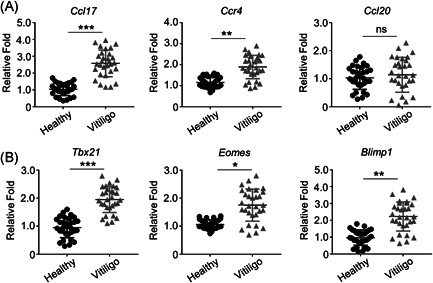
Vitiligo patients highly express the chemokines and their receptors in the skin. (A) The expression of indicated chemokines and their receptors in skin biopsies from vitiligo patients compared to age‐ and site‐matched controls by qRT‐PCR (*n* = 30). (B) The expressions of CD8 T cell‐specific transcriptional factors were measured by qRT‐PCR (*n* = 30). All data are presented as fold relative to the Actb mRNA level. Data are presented as mean ± SEM values and representative of at least three independent experiments. Statistical analyses represent variations in experimental replicates. *p < .05; **p < .01; ***p < .005 using one‐way ANOVA. mRNA, messenger RNA; qRT‐PCR, quantitative reverse transcription polymerase chain reaction

We initially examined CCL17 and CCR4 messenger RNA (mRNA) levels in skin samples from lesion area and its surrounding normal areas of 30 vitiligo patients. CCL17 and CCR4 levels in skin lesions were significantly increased compared with that in surrounding normal areas, while there was no obvious difference in CCL20 levels (Figure 1A). The loss of melanocytes and the infiltration of a large number of CD8^+^ T lymphocytes are often seen in the skin lesions of patients with vitiligo.^8^ Therefore, we detected the expression of key transcription factors for CD8^+^ T cell activation. Tbx21, Eomes, and Blimp1 mRNA levels in skin lesions were much higher than in surrounding normal areas (Figure 1B). Taken together, our results suggest that the CCL17‐CCR4 axis and CD8^+^ T cell activation are critical in vitiligo.

### 3.2 CCL17 is essential for the progression of vitiligo in mice

**Figure 2:**
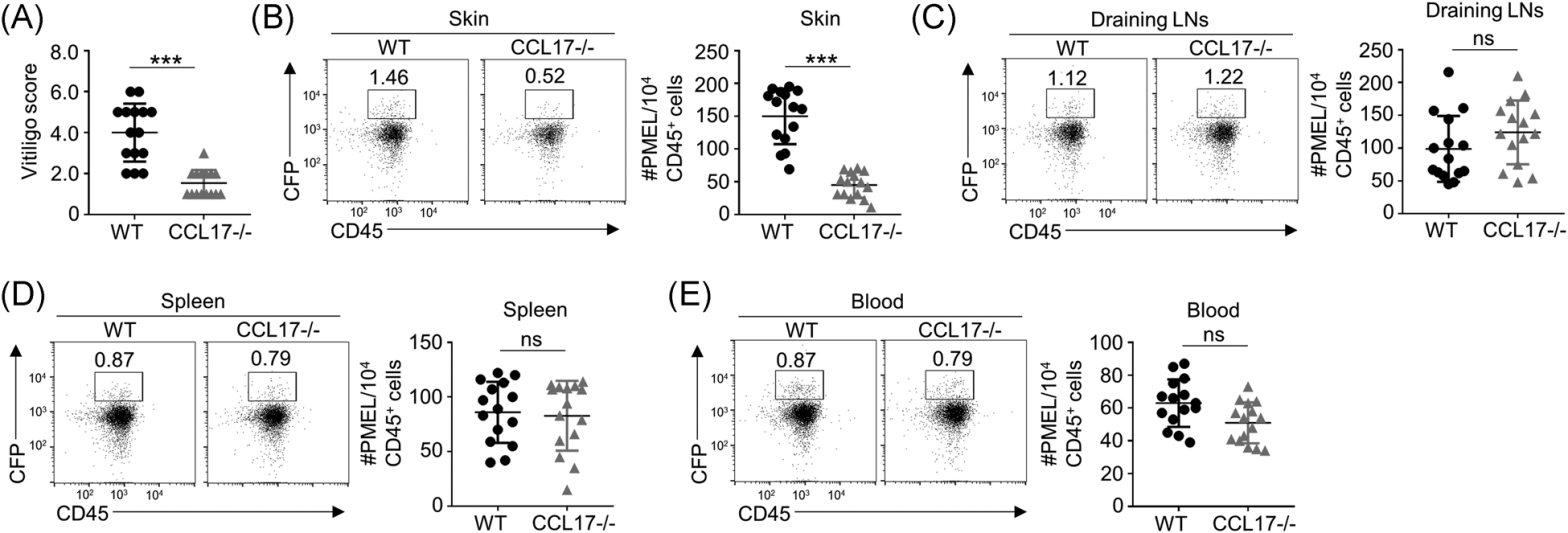
CCL17 is essential for vitiligo progression in mice. (A) The disease scores of control (WT) and CCL17‐deficent (CCL17^−/−^) mice (n = 15). (B) The cell numbers of CFP‐PMEL CD8^+^ T cells in the ear skin of WT or CCL17^−/−^ mice were shown by flow cytometry (*n* = 15). (C–E) The cell numbers of CFP‐PMEL CD8^+^ T cell showed no significant difference between WT and CCL17^−/−^ mice in lymph node, spleen, and blood (*n* = 15). Data are presented as mean ± SEM values and representative of at least three independent experiments. Statistical analyses represent variations in experimental replicates. ***p < .005 using one‐way ANOVA

To investigate whether CCL17 is critical for depigmentation, we adoptively transferred CFP‐PMEL CD8^+^ T cells into wild type (WT) or CCL17‐deficient (CCL17^−/−^) KRT14‐Kitl*4XTG2Bjl (Krt14‐Kitl*) mice to induce vitiligo. We observed that the disease scores of CCL17^−/−^ mice were dramatically lower than those of WT mice (Figure 2A). Flow cytometry showed CFP‐PMEL CD8^+^ T cell accumulation in various tissues. Interestingly, CCL17^−/−^ mice showed a clear decrease in CFP‐PMEL CD8^+^ T cell number in skin (Figure 2B), but did not affect the number in other tissues and organs, such as draining lymph nodes (Figure 2C), spleen (Figure 2D), and blood (Figure 2E). Our results suggest that CCL17 is necessary for CD8^+^ T cell recruitment to skin.

### 3.3 CCR4 essential for the progression of vitiligo in mice

**Figure 3:**
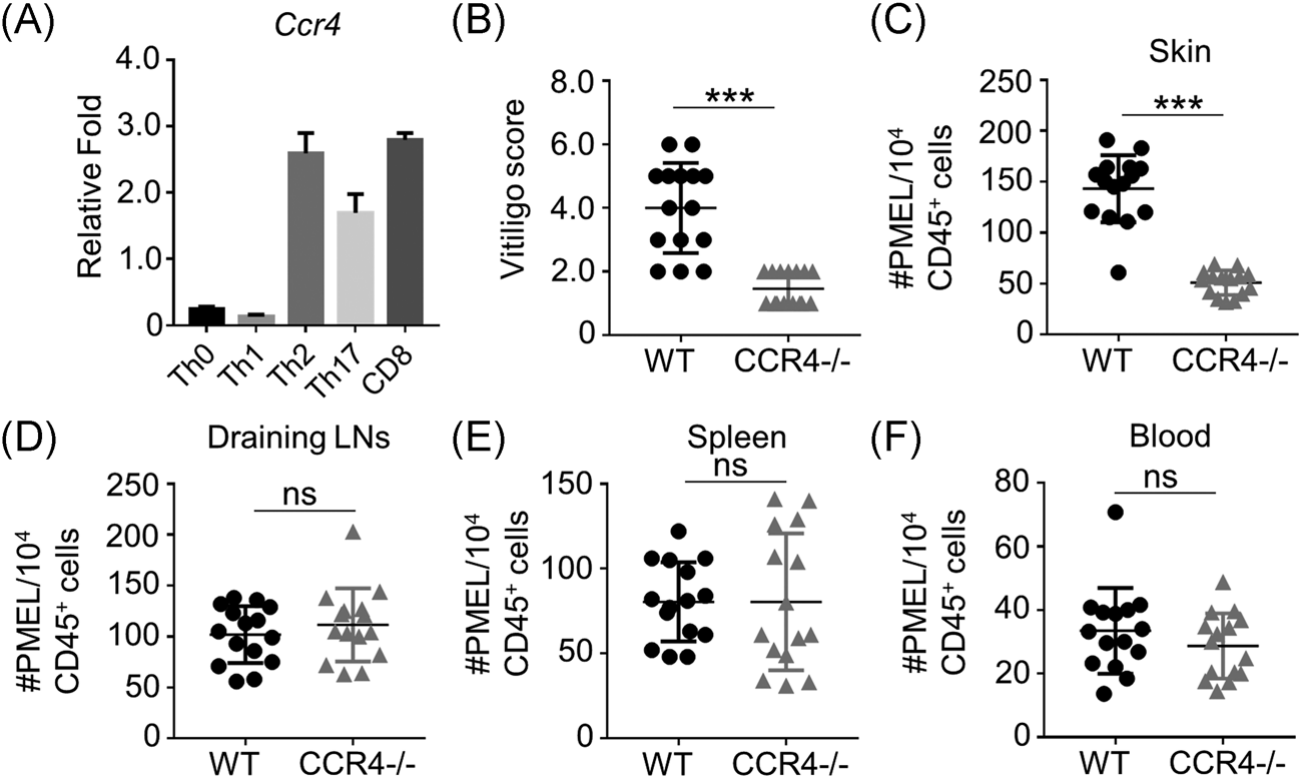
CCR4 is also essential for the onset of vitiligo in mice. (A) The expressions of indicated CCR4 in distinct T cell subpopulations were shown by qRT‐PCR (n = 4). (B) The disease scores of control (WT) and CCR4‐deficent (CCR4^−/−^) mice (*n* = 15). (C) The cell numbers of CFP‐PMEL CD8^+^ T cells in the ear skin of WT or CCL17^−/−^ mice were shown by flow cytometry (*n* = 15). (D–E) The cell numbers of CFP‐PMEL CD8^+^ T cell showed no significant difference between WT and CCR4^−/−^ mice in lymph node, spleen, and blood (*n* = 15). Data are presented as mean ± SEM values and representative of at least three independent experiments. Statistical analyses represent variations in experimental replicates. ***p < .005 using one‐way ANOVA

To further confirm the role of CCR4 as the receptor of CCL17, we compared CCR4 expression levels in various T cell subtypes. The results showed that CCR4 expression levels were higher in Th2, Th17, and CD8^+^ T cells when compared with in Th0 and Th1 cells (Figure 3A). To investigate whether CCR4 is critical for depigmentation, we adoptively transferred CFP‐PMEL CD8^+^ T cells (WT) or CCR4‐deficient CFP‐PMEL CD8^+^ T cells (CCR4^−/−^) into KRT14‐Kitl*4XTG2Bjl (Krt14‐Kitl*) mice to induce vitiligo. By evaluating the degree of depigmentation, we observed that CCR4^−/−^ mice exhibited significantly lower disease scores than WT mice (Figure 3B). Similar to the function of CCL17, the absence of CCR4 also significantly declined CFP‐PMEL CD8^+^ T cell number in skin (Figure 3C), but did not affect the number in other tissues and organs, such as draining lymph nodes (Figure 3D), spleen (Figure 3E), and blood (Figure 3F). So, CCL17‐CCR4 axis is necessary for CD8+ T cell recruitment to skin.

### 3.4 Neutralization of CCR4 reverses depigmentation in vitiligo mice

**Figure 4:**
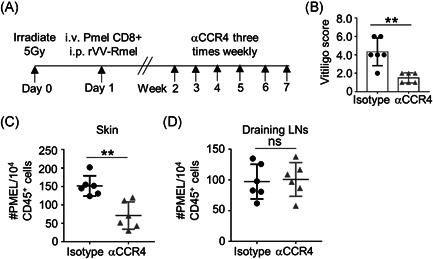
Blocking CCR4 reverses depigmentation in vitiligo mice. (A) The study design of CCR4 blockade. (B) The disease scores of mice administered with isotype control antibody (isotype) or neutralizing antibody (αCCR4) (*n* = 6). (C) The cell numbers of CFP‐PMEL CD8^+^ T cells in the ear skin of Isotype or αCCR4 mice were shown by flow cytometry (*n* = 6). (D) The cell numbers of CFP‐PMEL CD8^+^ T cell showed no significant difference between Isotype and αCCR4 mice in lymph node (*n* = 6). Data are presented as mean ± SEM values and representative of at least three independent experiments. Statistical analyses represent variations in experimental replicates. **p < .01 using one‐way ANOVA

To test the efficacy of CCR4 blockade as a treatment of vitiligo, we administered CCR4 neutralizing antibody or isotype control antibody to vitiligo mice (Figure 4A). Our results showed that mice treated with CCR4 neutralizing antibody exhibited significantly lower disease scores than mice treated with isotype control antibody (Figure 4B). Consistent with previous results, targeting CCR4 significantly inhibited CFP‐PMEL CD8^+^ T cell recruitment to skin (Figure 4C), while keeping T cell number unchanged in draining lymph nodes (Figure 4D). Our results indicate that neutralization of CCR4 reverses depigmentation in vitiligo mice.

## 4 DISCUSSION

Vitiligo, characterized by localized or generalized skin depigmentation and the formation of leukoplakia, is a relatively common pigmented disease.^18^ Its pathogenesis has not yet been fully elucidated. Here, we reported that the elevated expression of CCL17 and CCR4 is essential for the progression of vitiligo utilizing a mouse model. Blocking CCR4 affects the ability of CD8^+^ T cells migration to skin, while keeping the number of T cells in most tissues and organs unchanged. Our research suggests that targeting the CCL17‐CCR4 axis may be a new way of treating vitiligo.

The migration of T cells from peripheral blood to the skin is not a random process.^19^ The interaction of T cell homing receptor (cutaneous lymphocyte‐associated antigen [CLA]) and its ligand adhesion molecule E‐selectin could mediate T cell homing from peripheral blood to the skin.^20^ The increased level of CLA in CD8^+^ T cells of the skin lesions could promote the chemotaxis and migration of CD8^+^ T cells in the peripheral circulation to the skin, which in turn leads to skin inflammation.^21^ Van den Boorn et al.^22^ examined the cell activity at the effect phase around the skin lesion in a skin graft model and found that the autologous normal skin graft tissue was infiltrated by CD8^+^ T cells, which could kill the melanocytes. They also found that the apoptotic area of melanocytes is accompanied by the damage of keratinocytes. However, the effector T lymphocytes around the skin lesions could not induce the apoptosis of the keratinocytes in the area where the melanocytes are missing, suggesting that CD8^+^ T cells around the skin lesions have specific killing effects on melanocytes. Studies have shown that CD8^+^ T cells in patients with vitiligo express skin homing receptors and skin lymphocyte‐related antigens, thereby further mobilizing peripheral blood T cells to migrate to skin lesions.^23^ High levels of Melan‐A/MART‐1 specific CD8^+^ T cells are detected in the peripheral blood and skin lesions of patients with vitiligo.^24^ These cells exhibit strong cytotoxicity and skin homing ability in in vitro experiments, and could kill melanocytes by secreting granzyme B and perforin.^24^ In experiments with animal models of vitiligo, researchers found that T cells kill melanocytes by recognizing the H2‐Kb polypeptide of the melanocyte‐related antigen tyrosinase‐related protein 2 (TRP2).^25^ In the study of the vitiligo mouse model induced by TRP2‐180 peptide immunization, it was found that the number of TRP2‐180 specific CD8^+^ T cells is proportional to the area of depigmentation in mice.^26^ Another study showed that patients with active vitiligo revealed an increase of perforin and granzyme‐B in CLA^+^CD8^+^ T cells, suggesting that CD8^+^ T cells play a leading role in the destruction of melanocytes in vitiligo.^14^ In addition, patients with melanoma may experience vitiligo during immunotherapy. Studies have found that this phenomenon is positively correlated with the prognosis of melanoma. Therefore, the killing effect of melanocyte‐specific CD8^+^ T cells on melanocytes could be used as a possible clinical strategy to treat melanoma.^27^ All of the above suggested that CD8^+^ T cells have specific cytotoxicity and skin homing ability, which might play a leading role in the destruction of melanocytes in vitiligo. In the present study, we demonstrated that the expressions of key transcription factors for CD8^+^ T cell activation were remarkably upregulated in skin lesions compared with that in surrounding normal areas, which further confirmed the role of CD8^+^ T cells in the skin lesions of vitiligo.

The high levels of CCR4 on Th2 cells and skin‐homing T cells expressing CLA suggest that CCR4 is involved in skin‐associated immune responses.^28^, ^29^ Although CCR4 is considered as a marker of Th2 cells and mainly expressed on CD4^+^ T cells, CCR4 was also found to be expressed on CD8^+^ T cells.^30^ Zhang et al.^14^ reported that the proportion of CCR4 on CD8+T cells in peripheral blood of active vitiligo patients were remarkably higher than that of stable vitiligo patients or healthy controls. Paradoxically, Yang et al.^31^ revealed that the expressions of CCR4 showed no significant difference between halo nevus, lesions of vitiligo, and healthy controls. In this paper, we examined the expression of CCL17 and CCR4 in the lesion area and its surrounding normal areas of vitiligo 30 patients. A remarkable increase of CCL17 and CCR4 was observed in the skin lesions, suggesting that they play a critical role in depigmentation. Notably, our findings used the normal area around the skin lesion as a control instead of the tissues of normal volunteers for the first time, which might be the cause of the different results. Furthermore, we demonstrated that CCR4 is enriched in Th2, Th17, and CD8^+^ T cells. Depletion of CCL17 or CCR4 significantly inhibited the progress of depigmentation in mouse model induced by transferring CFP‐PMEL CD8^+^ T cells. Therefore, the elevated CCL17‐CCR4 signaling in CD8^+^ T cells of the skin lesions might be critical in vitiligo. Therefore, we further blocked the CCR4 by neutralizing antibody and found that targeting CCR4 significantly inhibited depigmentation and the recruitment of CD8^+^ T cells to the skin.

The expression of CCL17 and CCR4 has also been shown in Tregs^32^ and dendritic cells (DCs).^33^ Therefore, it is important to determine whether the difference in the progression of vitiligo is caused by the balance between effector T cells and Treg cells or the depletion of immunogenic DCs. In this article, the mouse vitiligo model was induced by adoptive transfer of PMEL CD8^+^ T cells. Thus, the data (Figure 3) indicated that CCR4 mainly functioned in CD8^+^ T cells, rather than in Treg or DCs, even though increased CCR4 expression was indeed found in both types of cells. However, it will be interesting to evaluate the role of DCs or Treg‐specific CCR4 in our future work.

In conclusion, our results demonstrated the high expression of CCL17, CCR4 and the elevated activation of CD8^+^ T cells in skin lesions of vitiligo patients. CCL17^−/−^ and CCR4^−/−^ mice exhibited significantly lower degree of depigmentation and CD8^+^ T cells accumulation in skin. CCR4 expression was enriched in Th2, Th17 and CD8^+^ T cells. Blockade of CCR4 by neutralizing antibody revealed a same trend as CCL17^−/−^ and CCR4^−/−^ mice. Our findings suggest that blocking the CCL17‐CCR4 signaling might suppress the depigmentation and CD8^+^ T cells migration to skin, thereby alleviating the progression of vitiligo.

## CONFLICT OF INTERESTS

The authors declare that there are no conflict of interests.

## AUTHOR CONTRIBUTIONS

He Li, Congpin Wang, Xiaoqing Li, Yinghui Kong, and Weiguo Sun performed the experiments, analyzed and interpreted the data. He Li, Congpin Wang, and Weiguo Sun wrote the manuscript. All authors read and approved the final manuscript.

## ETHICS STATEMENT

Animal studies were approved by the ethics commitment of the Affiliated Huaian No. 1 People's Hospital of Nanjing Medical University.

## Data Availability

Data could be obtained upon request to the corresponding author.
